# Antimicrobial metal-based nanoparticles: a review on their synthesis, types and antimicrobial action

**DOI:** 10.3762/bjnano.11.129

**Published:** 2020-09-25

**Authors:** Matías Guerrero Correa, Fernanda B Martínez, Cristian Patiño Vidal, Camilo Streitt, Juan Escrig, Carol Lopez de Dicastillo

**Affiliations:** 1 Center of Innovation in Packaging (LABEN), University of Santiago de Chile (USACH), Obispo Umaña 050, 9170201 Santiago, Chile; 2 Center for the Development of Nanoscience and Nanotechnology (CEDENNA), 9170124 Santiago, Chile; 3 Department of Physics, University of Santiago de Chile (USACH), Av. Ecuador 3493, 9170124 Santiago, Chile

**Keywords:** antimicrobial mechanism, antimicrobial nanoparticles, metallic nanoparticles, nanoparticle synthesis, nosocomial infections

## Abstract

The investigation of novel nanoparticles with antimicrobial activity has grown in recent years due to the increased incidence of nosocomial infections occurring during hospitalization and food poisoning derived from foodborne pathogens. Antimicrobial agents are necessary in various fields in which biological contamination occurs. For example, in food packaging they are used to control food contamination by microbes, in the medical field the microbial agents are important for reducing the risk of contamination in invasive and routine interventions, and in the textile industry, they can limit the growth of microorganisms due to sweat. The combination of nanotechnology with materials that have an intrinsic antimicrobial activity can result in the development of novel antimicrobial substances. Specifically, metal-based nanoparticles have attracted much interest due to their broad effectiveness against pathogenic microorganisms due to their high surface area and high reactivity. The aim of this review was to explore the state-of-the-art in metal-based nanoparticles, focusing on their synthesis methods, types, and their antimicrobial action. Different techniques used to synthesize metal-based nanoparticles were discussed, including chemical and physical methods and “green synthesis” methods that are free of chemical agents. Although the most studied nanoparticles with antimicrobial properties are metallic or metal-oxide nanoparticles, other types of nanoparticles, such as superparamagnetic iron-oxide nanoparticles and silica-releasing systems also exhibit antimicrobial properties. Finally, since the quantification and understanding of the antimicrobial action of metal-based nanoparticles are key topics, several methods for evaluating in vitro antimicrobial activity and the most common antimicrobial mechanisms (e.g., cell damage and changes in the expression of metabolic genes) were discussed in this review.

## Review

### Introduction

In the last decades, the search for new antimicrobial substances against microbial contamination has been the focus of many research fields, in public and private research centers, in order to reduce nosocomial infections and foodborne diseases. The elimination of pathogenic microorganisms, such as bacteria, fungi, and yeast, in order to avoid health issues has been a major goal in these fields. There are two terms that can define the antibacterial efficiency of a given compound: An agent is considered “bacteriostatic” if it delays the bacterial growth, maintaining the initial growth phase for a longer period of time. An antibacterial agent can also be “bactericidal” if it completely inhibits and kills the bacteria. However, the bacteriostatic and bactericidal effects exhibited by the compounds during in vitro experiments depend on several factors, including bacterial density, test duration, growth conditions, and the reduction in bacteria concentration. For these reasons, in many studies the compounds are better described as substances with excellent antibacterial properties, since they can exhibit both effects [1]. Furthermore, the antibacterial effectiveness of most compounds differs depending on the type of bacteria exposed to these compounds. Gram-positive and Gram-negative bacteria, for example, are categories widely studied due to their different cellular structure which might affect the antimicrobial effectiveness of a given compound. Gram-positive bacteria have a thicker peptidoglycan layer, whereas Gram-negative bacteria contain a thin peptidoglycan layer and an outer membrane [2].

The presence of mold and yeast, mainly in food sources, has also attracted research interest [3–4]. Although several solutions have been proposed, microorganism incidence will continue to increase and will remain a complicated challenge to overcome. Due to the reoccurrence of infections, microorganisms have become resistant to antibiotics as a result of inherent genetic changes caused either by misuse or excessive use of drugs and antimicrobial agents, which significantly impacts the public health system. Thus, the research and development of a new generation of innovative and effective antimicrobial agents have become an urgent need. In this search, the scientific community has been focusing on the study of nanomaterials, mainly metal-based nanoparticles (NPs), to test their antimicrobial properties and feasibility to eradicate contamination sources and diseases [5]. The chemical, physical, and biological properties of NPs have been improved on the nanometer scale regarding their surface area, size, distribution, and morphology [6–8]. Research evidence shows that antimicrobial properties clearly depend on the synthesis method used to obtain the NPs. These synthesis procedures can be classified into physical, chemical, and biological methods [9]. In general, physical methods have the highest economic and energetic costs [10]. Therefore, research has leaned towards chemical synthesis methods which are able to produce a large number of NPs in a shorter period of time [11]. The research in this area, especially the “green synthesis” methods, has undoubtedly received significant attention regarding their low environmental impact compared to other procedures [12–13]. There are different routes in which the green synthesis methods are applied through the use of microorganisms and plants in a safe, efficient, and profitable manner [14].

Different types of metal-based NPs have demonstrated antimicrobial activity over the last years. Several metal and metal oxide NPs, such as silver, copper, zinc oxide, titanium oxide, copper oxide, and nickel oxide NPs, are known to display antimicrobial activity [15–17] that depends on their composition, surface modification, intrinsic properties and the type of targeted microorganism [18]. A special category of metallic NPs is superparamagnetic iron-oxide nanoparticles (SPIONs) (e.g., magnetite (Fe_3_O_4_) and maghemite (γ-Fe_2_O_3_) NPs) whose antimicrobial activity increases upon the application of an external magnetic field [19]. An interesting strategy to increase the antimicrobial efficiency of metal-based nanoparticles is the use of silica and carbon compounds as delivery systems [20].

The broad range of metal-based nanoparticles, the types of NP synthesis, and their antimicrobial activity were further explored in this review. Different methods to analyze the efficiency of the antimicrobial activity of metal-based NPs have also been discussed [21–22]. In addition, some particular NP antibacterial mechanisms that affect the different essential structures of the microorganisms were discussed, such as the induction of oxidative stress, the release of metal ions and the non-oxidative damage.

### Synthesis of antimicrobial nanoparticles

Over the last years, techniques for synthesizing antimicrobial nanoparticles have advanced significantly due to their use in both biomedical and industrial applications. The properties of nanoparticles strongly depend on the synthesis technique used, which determines both the morphology and size of the NPs. As mentioned earlier, the synthesis methods can be grouped into physical, chemical, and biological (also called green synthesis) methods, which will be discussed in the next subsections.

#### Physical methods

Examples of physical methods used to synthesize NPs are the evaporation/condensation method, magnetron sputtering, mechanochemical processing (MCP), microwave-thermal method, photoreduction process, and pulsed laser ablation, among others.

The evaporation/condensation method allows for nanoparticles to be synthesized directly from a metal source. Jung et al., for example, obtained Ag NPs in various sizes, ranging from 6 to 21 nm. However, this technique has the disadvantage of consuming a significant amount of energy and it requires a large lab space for the experimental setup [23]. Magnetron sputtering is a high-rate vacuum-coating technique generally used to synthesize films, multilayer or hybrid systems based on substrate coating. For example, Piedade et al. obtained ZnO, ZnO–C and ZnO–Cu films with thickness values ranging from 385 to 1635 nm. In addition, Galstyan et al. produced coated sheets of reduced graphene oxide (rGO) which formed a composite compound containing ZnO [24–25]. One method that facilitates the large-scale production of nanoparticles is the MCP technique. This method is based on a chemical exchange reaction that occurs due to the heat and pressure at which the ball mills are subjected to in a grinder. By using this technique, Kim et al. have discussed how ZnO NPs can be developed with sizes ranging from 20 to 30 nm [26]. The microwave-thermal method [27–30] allows small particles to be obtained with a narrow size distribution from different materials in a fast, safe, and environmentally friendly way. This technique allows for the synthesis of Ag [27], CuO [28], and MgO [29] NPs with sizes ranging from 1 to 25 nm. In addition, with this technique it is possible to control the geometry of the nanoparticles in order to obtain squared and polyhedral-shaped nanoparticles, for example, without compromising their size [30]. Another interesting technique used is the very slow ultraviolet irradiation photoreduction process in which the morphology of the nanoparticles can be controlled based on the cation concentration and the irradiation time. For example, while Tan et al. [31] obtained spherical silver nanoparticles, Zhou et al. [32] obtained plate-like triangles. Another method used is the pulsed laser ablation technique which is used to synthesize colloidal solutions of Ag [33], Au [34], MgO [35], and ZnO [36] NPs, among others, via a high-power pulsed laser beam that hits a target of the material of choice.

In this context, several physical methods have been used to synthesize nanoparticles, and the most relevant ones, along with the typical resulting particle sizes, are listed in Table 1. Depending on the preparation conditions, the size, length, and diameter of the nanostructures can be adjusted in order to control the physical properties of the NPs.

**Table 1:** Physical methods used for synthesizing NPs and some examples of materials used in the synthesis processes.

Fabrication technique	Material	Size and/or morphology

evaporation/condensation method [23]	Ag	6–21 nm NPs

magnetron sputtering [24]	ZnO	455–1635 nm thin films
	ZnO–C	385 nm thin films
	ZnO–Cu	705 nm thin films

magnetron sputtering, anodization and thermal treatment [25]	rGO–ZnO	hybrid nanostructure

MCP [26]	ZnO	20–30 nm NPs

microwave-thermal method [27–30]	Ag	10 nm NPs
	CuO	1–25 nm NPs
	MgO	10 nm NPs
		4 nm squared and polyhedral-shaped NPs

photoreduction process [31–32]	Ag	33–66 nm NPs
	Au	15 nm plate-like triangles

physical vapor deposition (PVD) [26]	ZnO	8–75 nm NPs

pulsed laser ablation technique [33–37]	Ag	35 nm NPs
		40 nm NPs
	Au	spherical NPs
	MgO	controllable size, shape, and composition
	ZnO	porous nanostructures

#### Chemical methods

A few examples of chemical methods that have been used to synthesize nanoparticles are the atomic layer deposition method, chemical reduction method, chemical vapor deposition, electrochemical anodization method, hydrolysis, hydrothermal method, precipitation–hydrothermal method, reverse micellar route, sol–gel method, solution-based synthesis, solvothermal synthesis, and the sonochemical method. The most relevant ones, along with the typical resulting particle sizes, are listed in Table 2.

**Table 2:** Chemical methods used for synthesizing NPs and some examples of materials used in the synthesis processes (carbon nanotubes (CNTs), multiwall carbon nanotubes (MWCNTs)).

Fabrication technique	Material	Size and/or morphology

atomic layer deposition (ALD) [38–41]	TiO_2_	(*r* = 345 nm, *t* = 17 nm)^a^ hollow nanospheres
		(*d* = 165 nm, *t* = 20 nm)^a^ nanotubes
	ZnO	(*d* = 180 nm, *t* = 60 nm)^a^ nanotubes
		(*d* = 80 nm, *t* = 20 nm, *L* = 70 µm)^a^ nanotubes

chemical reduction method [42]	Ag	20–80 nm well-dispersed spherical NPs

chemical vapor deposition (CVD) [43–44]	CNTs (MWCNTs)	(*d* = 30 nm, *L* = 70 µm)^a^ nanotubes
	graphene/Ag nanowires	hybrid coating

cryochemical synthesis [45]	Ag	20–150 nm NPs

electrochemical anodization method [46–47]	Ag	spherical NPs
	ZnO	(*d* = 10–75 nm, *L* = 1 µm)^a^ NPs forming elongated aggregates with a chain-like morphology

hydrolysis [48–49]	CeO_2_	≈7 nm ellipsoidal monocrystallites

reverse micellar route [50–52]	MgO	8–10 nm NPs
	TiO_2_	10–20 nm NPs
	ZnO	(*d* ≈ 6 µm)^a^ flower-like microstructures and (*d* ≈ 1 µm, *L* ≈ 4 µm)^a^ tube-like nanostructures

sol–gel method [53–57]	MgO	35–50 nm assembled NPs with a mesoporous structure
		NPs with different shapes and sizes
	N–Ag–TiO_2_–ZnO	300–500 nm nanocages
	TiO_2_	8–40 nm NPs
		4–10 nm NPs

solvothermal synthesis [58–59]	Y_2_O_3_	140 nm square platelets and (*d* = 30 nm, *L* = 150 nm)^a^ rods
	ZnO	different morphologies and sizes

hydrothermal (HT) method [60–62]	CeO_2_	25–50 nm spherical NPs
	MgO	20–200 nm lamellar NPs
	ZnO	(*d* = 100 nm, *L* = 3 µm) nanorods

sonochemical method [63–65]	Ag	20 nm spherical NPs
	CeO_2_	≈20 nm cubic-shaped NPs
	CuO	5–10 nm spherical NPs

solution-based synthesis [66–68]	Au	150–1000 nm nanoporous NPs
		1–3 nm spherical NPs
	CeO_2_	≈100 nm NPs

^a^NP parameters: *r*: radius; *d*: diameter; *t*: thickness; *L*: length.

**Figure 1:**
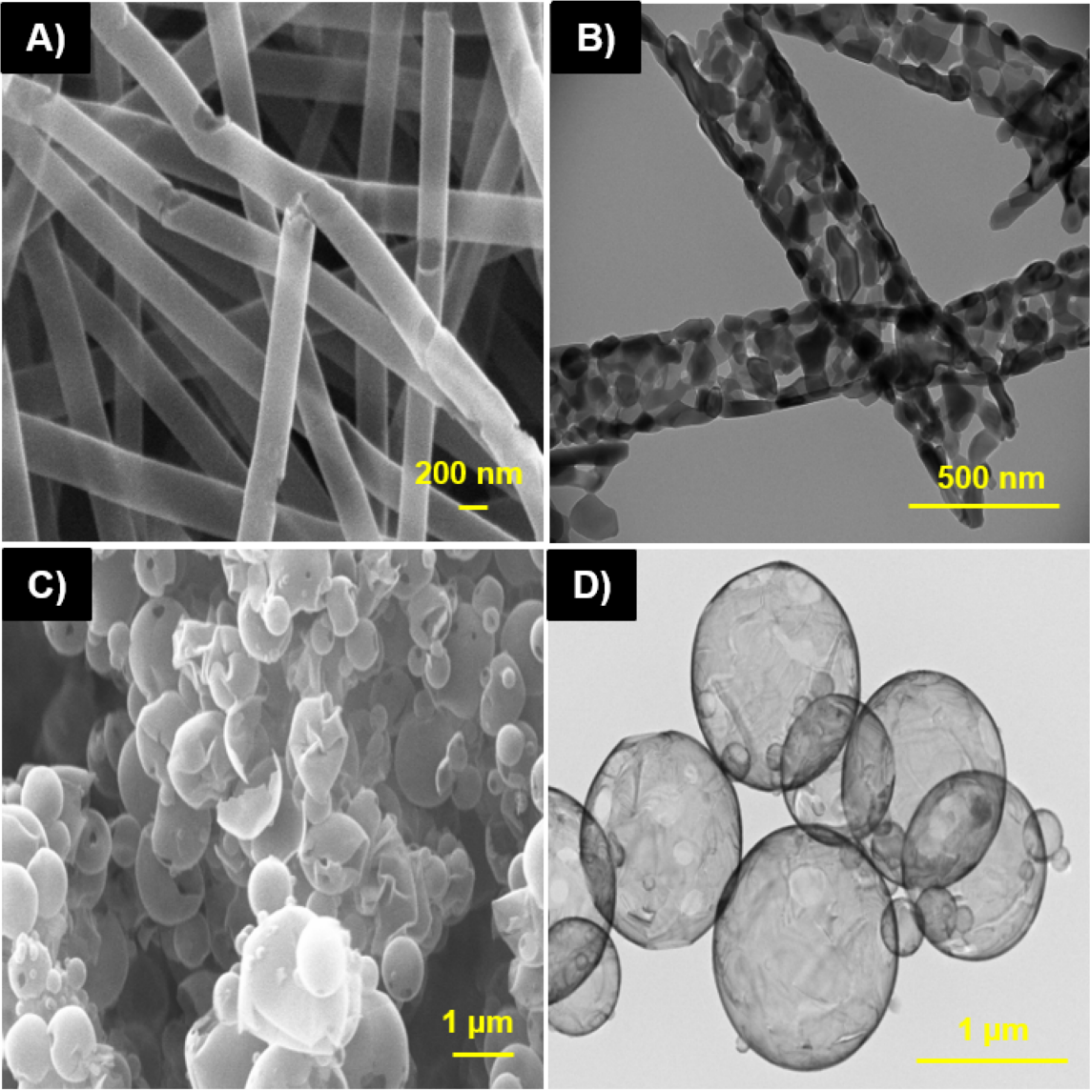
SEM (A) and TEM (B) images of hollow TiO_2_ nanotubes. SEM (C) and TEM (D) micrographs of hollow TiO_2_ nanospheres.

The atomic layer deposition method is employed to grow metal oxide and metallic three-dimensional nanostructures using porous alumina membranes [41], electrostatically spun nanofibers [39–40] or electrosprayed spherical particles [38] as templates. As Figure 1 shows, Lopez de Dicastillo et al. (2018, 2019) have recently developed hollow titanium dioxide nanotubes and nanospheres through the deposition of tetrakis(dimethylamide) titanium and water, as precursors, on polymeric structures obtained via electrospinning. The resulting hollow nanotubes and nanospheres had thickness values of approximately 20 and 17 nm, respectively [38–39]. ALD has been recognized as a key technique used to deposit thin films on structures with complex geometries, allowing for the synthesis of nanostructures without shadowing effects and with a high aspect ratio, such as nanotubes with diameters ranging between 80 and 180 nm and length values that can reach several tens of micrometers.

The chemical reduction method was initially proposed by Michael Faraday in 1856–1857 while investigating the properties of colloidal gold. This method generally uses a precursor, a reducing agent, and a stabilizer or protective agent [42]. Occasionally, a catalyst can be added to accelerate the reaction, as well as a solvent, which can favor the interaction of the chemicals. As an example, we highlight the work of Wang et al. in which well-dispersed spherical nanoparticles with sizes ranging from 20 to 80 nm were synthesized [42]. The chemical vapor deposition method is a technique in which the substrate is exposed to one or more volatile precursors, which react to and/or decompose on the substrate surface to produce the desired thin film deposit. For example, Zhao et al. [44] obtained graphene-wrapped Ag nanowires using the chemical vapor deposition method in order to investigate their broad-spectrum and robust antimicrobial properties. The cryochemical synthesis method includes a simultaneous evaporation of a metallic and a volatile component (e.g., an organic monomer), followed by co-condensation of the vapors on the cold surface of the vacuum reactor. Sergeev et al. [45] obtained Ag nanoparticles with sizes ranging from 20 to 150 nm using this technique. The electrochemical anodization method is based on the reactions that occur between the electrode and the electrolyte. In this method, electricity is used as the driving or controlling force. The main advantages of using electrochemical techniques include using vacuum-free systems, simple operation procedures, high flexibility, low cost, low contamination (pure product), and the fact that it is an environmentally friendly process. In addition, with this technique one can control the morphology of the synthesized nanoparticles. For example, while Johans et al. [46] obtained spherical Ag nanoparticles, Galstyan et al. [47] synthesized nanoparticles in the form of elongated aggregates with a chain-like morphology. The hydrolysis technique, which involves the reaction of an organic chemical with water, has been used to obtain ellipsoidal monocrystallites of CeO_2_ with an average size of ≈7 nm [48–49]. The reverse micelle technique, also known as the microemulsion technique, is used to synthesize nanoparticles of various materials with different morphologies and sizes. For example, the technique has been used to obtain ultrafine MgO nanoparticles (8–10 nm) [50], TiO_2_ nanoparticles (10–20 nm) [51], and even flower-like microstructures (diameter ≈6 µm) and microtubes (diameter ≈1 µm and length ≈4 µm) [52]. The sol–gel technique has been widely used to synthesize nanoparticles since it is a simple and relatively fast technique. With this technique it is possible to fabricate TiO_2_ NPs smaller than 10 nm [56–57] and MgO NPs with sizes ranging from 35 to 50 nm [53–54]. Furthermore, it has been used in the synthesis of N–Ag–TiO_2_–ZnO nanocages with diameters ranging from 300 to 500 nm [55].

The solvothermal synthesis method is a technique used to prepare a variety of materials, such as metals, semiconductors, ceramics, and polymers. In this process, the chemical reaction takes place in a sealed vessel where solvents are brought to a temperature well above their boiling point, facilitating the interaction of the precursors during synthesis. Some examples are Y_2_O_3_ [58] and ZnO [59] nanoparticles with different morphologies and sizes. When water is used as the solvent, the method is called the hydrothermal technique, which is an easy and convenient method for growing NPs. By varying the synthesis parameters, a variety of nanostructures, such as spherical CeO_2_ NPs [61], lamellar MgO NPs [62], and even ZnO nanorods [69] can be obtained by using this method. In the sonochemical synthesis method, a high-intensity ultrasound produces an acoustic cavitation that can be used for the production or modification of a wide range of nanostructured materials. Some examples are spherical Ag [63] and CuO NPs [65], and square-shaped CeO_2_ NPs [64]. The main advantage of this technique is the simplicity in maintaining the operating conditions (ambient conditions) and controlling the NP size.

#### Green synthesis

Generally, the procedures for obtaining nanoparticles are physical and/or chemical methods in which a precursor material reacts with reducing agents, as mentioned earlier. Nevertheless, both methods have been proven to be harmful for the environment and living organisms [70–71]. Moreover, the physical-based synthesis methods require expensive equipment, high temperature and high pressure, which makes it an unprofitable and unscalable method [10]. On the other hand, the chemical methods use and generate toxic chemicals that can cause dangerous effects to the environment, in addition to being cytotoxic and carcinogenic [72]. Several toxic chemicals adhered to the particles synthesized through these methods have been identified [13,73]. For these reasons, an interest in environmentally friendly nanoparticle synthesis methods, also called "green synthesis" or "nanoparticle biosynthesis" methods, has arisen. In addition to their ecologically friendly nature, these techniques also present a higher performance, less energy costs (temperature and pressure), and they are profitable, biocompatible, safe, and easy to expand on a larger scale [72,74].

With the advance of nanotechnology, the number of antibiotic-resistant bacteria has also increased, including strains that are resistant to more than 100 different types of antibiotics [75]. This problem, in addition to the environmental concerns, has inspired many researchers to develop ecologically friendly biosynthetic nanoparticles as antimicrobial agents. Several ecological routes have been investigated, focusing on the search for natural resources. Methods based on the biological synthesis of nanoparticles through the usage of plant extracts [76–77], raw materials from fruits and vegetables [78], algae [79], bacteria, fungi [80], and residues [81] have been reported. The products obtained from these methods are called biogenic NPs [82], whereas the biological organisms used in these processes are called biological nanofactories, which can release proteic substances that are able to chemically reduce metal ions [83]. It is important to highlight that all the biological components have a unique chemical structure, their own metabolic pathways, and different responses depending on the metallic ion used to control and modify the synthesis of NPs. The green synthesis of NPs can occur both intracellularly or in the extracellular milieu, where the biomolecules released from the cells are located. As an example of the latter, *Pseudomonas strutzeri* bacteria can successfully generate Ag NPs extracellularly [84]. Conversely, the bioreduction of iron followed by the precipitation of an oxide, which is subsequently transformed into FeO NPs, occurs through an intracellular synthesis pathway since the bacteria or fungi carry the ions to the intracellular space [85]. The use of plants presents some advantages over other production sources since phytochemicals can act as protecting and stabilizing agents, eliminating an additional step to prevent particle aggregation [86]. In addition, cell culture procedures are not necessary in this case, which allows for the large-scale synthesis of nanoparticles in a non-aseptic environment [87]. Furthermore, plant-based processes are cost-effective and safe for humans and the environment [10]. Different parts of the plants can be used in the green synthesis of NPs. For example, spherical copper NPs (≈5–20 nm) were obtained by using *Curcuma longa* tuber extract and copper acetate dehydrate. These Cu NPs demonstrated excellent antibacterial activity against Gram-negative bacteria (inhibition zone diameter of *E. coli*: 22 ± 0.86 mm) and Gram-positive bacteria (inhibition zone diameter of *B. subtilis*: 23 ± 0.9 mm) [88]. Bio-reduction of silver nitrate with *Parkia speciosa* leaf extract generated spherical Ag NPs with an average particle size of 31 nm [89]. A major antibacterial activity against *S. aureus* was followed by *B. subtilis*, *E. coli* and *P. aeruginosa.* By using latex extracted from an immature *Papaya carica* fruit and silver nitrate, spherical and highly stable Ag NPs were also obtained. The reduction in Gram-positive bacteria, such as *E. faecalis* and *B. subtilis*, was lower than the reduction in Gram-negative bacteria, such as *V. cholerae*, *P. mirabilis*, *E. coli*, and *K. pneumonia*.

ZnO NPs are of great interest because their synthesis is economical, safe and easy [72]. Vijayakumar et al. (2018) investigated the antimicrobial and antifungal effect of spherical ZnO NPs (30 nm) that were successfully synthesized using *Atalantia monofila* leaf extract [90]. The bactericidal effect against Gram-positive and Gram-negative bacteria was evaluated and the highest inhibition values were obtained for *B. subtilis* (inhibition zone diameter of 20 mm) and *K. pneumoniae* (inhibition zone diameter of 19 mm). On the other hand, the maximum inhibition zones for fungi were observed against *C. albicans* (inhibition zone diameter of 24 mm) followed by *A. niger* (inhibition zone diameter of 18 mm).

Over the last years, the replacement of plants by other biological samples, mainly bacteria and fungi, has increased. The antimicrobial activity of CuO NPs obtained from an *Actinomycete* bacteria in a copper sulfate aqueous solution was tested against a few pathogenic bacteria, such as *Staphylococcus aureus*, *Bacillus cereus*, *Proteus mirabilis*, *Edwardsiella tard*, *Aeromonas caviae*, *Aeromonas hydrophila* and *Vibrio anguillarum*. The results showed that the highest zone of inhibition was obtained for *B. cereus* (inhibition zone diameter of 25.3 mm), followed by *E. tard* (inhibition zone diameter of 22.6 mm), at a concentration of 100 µg/mL [91]. However, the synthesis of NPs using bacteria has several disadvantages, such as the high cost of culture media, the need for microbial screening, long process time, microbial contamination, and lack of control over the morphological characteristics. Due to these disadvantages, the attention towards using fungi to synthesize NPs has increased, and even common food-contaminating fungi, such as white rot fungi, are being considered. Studies have shown that the antimicrobial properties are dependent on the type of fungus used. Gudikandula et al. (2017) successfully obtained spherical and well-dispersed Ag NPs by using two different fungal strains (*Ganoderma enigmaticum* and *Trametes ljubarskyi*) and silver nitrate [92]. The generated NPs presented a size range varying between 15 and 25 nm and their antimicrobial activity was evaluated against eight pathogenic bacteria. Ag NPs obtained from *G. enigmaticum* fungi showed the greatest inhibition zone against *Staphylococcus* (KUCC 7) and *P. putida* (KUCCC 12) with an average diameter of 28 mm, while Ag NPs synthetized from *T. ljubarskyi* fungi had the highest inhibition zone against *B. subtilis* (MTCC 441) and *P. putida* (KUCCC 12) with an average diameter of 28 nm.

Another trend of the ecologically friendly approach to synthesize nanoparticles has been the use of biological and agricultural waste as reducing agent sources. Researchers have been focusing on this field that leads to one of the most ecologically friendly, sustainable, cost-efficient, and easy methods for synthesizing NPs. Recently, Soto et al. (2019) used fruit peel waste to synthesize silver nanoparticles with antimicrobial activity against foodborne pathogens [93]. Ibrahim et al. (2015) have also synthesized spherical silver nanoparticles (23.7 nm) by using banana peels. These Ag NPs have exhibited antimicrobial activity against microorganisms, showing larger inhibition zones against Gram-negative bacteria (*E. coli* and *P. aeruginosa*) when compared to Gram-positive bacteria (*B. subtilis* and *S. aureus*) [94].

### Types of metal-based antimicrobial nanoparticles

#### Metallic and metal-oxide nanoparticles

Since ancient times, metal-based materials, such as silver and copper, have been used as antimicrobial agents by the Egyptians, Persians, Greeks and Romans. Nowadays, due to efforts in nanotechnology, metallic NPs synthesized through different methods have drawn the most attention due to their functionality [95]. Specifically, metallic NPs provide strong and extended antimicrobial activity at smaller dosages against a broad range of microorganisms due to their dimensions and shapes [96]. Table 3 shows some examples of potential antimicrobial metallic NPs. Silver nanoparticles have been considered one of the most interesting antimicrobial metallic NPs due to their high efficiency against bacteria, fungi, and viruses. Their high antimicrobial activity enables use in pharmaceutical, food, fabric, and packaging industries [97–99]. Nanda et al. (2018) obtained Ag NPs through green synthesis methods by using different extracts of the fungus *Aspergillus tamarri* and AgNO_3_. The resulting Ag NPs showed a potential antimicrobial activity against *Candida albicans* and *Staphylococcus aureus* [100]. Spherical Ag NPs, with a diameter of 16 nm, showed antibacterial properties against the human pathogens *Escherichia coli* and *Pseudomonas aeruginosa* [101]. Chen et al. (2019) also developed an antimicrobial-based biocomposite containing Ag NPs with good antibacterial properties against *E. coli* and *S. aureus*, as shown by the disk diffusion method [102].

**Table 3:** Antimicrobial studies with metallic and metal-oxide NPs over the last years.

Nanoparticle material	Size and/or morphology	Microorganism	Antimicrobial activity method

Ag [98]	10–20 nm spherical	*S. aureus*, *P. aeruginosa*	dynamic contact
Ag [102]	8–16 nm spherical	*S. aureus*, *E. coli*	agar disk diffusion
Ag–ZnO [99]	flake-like aggregates	*S. aureus*, *Streptococcus*, *E. coli*, *Pseudomonas*, *Candida spp*	agar disk diffusion
Ag [101]	16 nm spherical	*S. aureus*, *E. faecalis*, *P. aeruginosa*, *E. coli*	dynamic contact
Ag [103]	80–90 nm rod-shaped	*S. aureus*, *P. aeruginosa*	dynamic contact
Cu [104]	20–100 nm	*S. aureus*	surface contact
Cu-loaded NaX zeolite [105]	2–4 µm spherical	*E. coli*	agar disk diffusion
ZnO coated by CuNPs [106]	5 nm needle-shaped and spherical	*E. coli*, *S. aureus*	agar disk diffusion
Cu [107]	6–9 nm	*B. subtilis*	dynamic contact
Cu [108]	25 nm spherical	*B. subtilis*, *C. perfringens*, *P. aeruginosa*, *E. coli*, *S. aureus*, *L. monocytogenes, C. tropicalis*, *F. verticillioides*	dynamic and surface contact
ZnO [109]	50–400 nm star-like	*B. subtilis, E. aerogenes*	surface contact
ZnO [110]	20–40 nm	*S. aureus*, *E. coli*	agar disk diffusion
ZnO [111]	120–400 nm	*B. subtilis*, *S. aureus*, *E. coli*, *P. aeruginosa*	dynamic contact
ZnO [112]	70 nm hexagonal	*L. plantarum*	surface contact
ZnO [113]	nanoflowers	*E. coli*	agar well diffusion
TiO_2_ [3]	20 nm spherical	*Lactobacilli Streptococci*, mold, yeast, coliform	surface contact
TiO_2_ [114]	nanotubes	*E. coli*	dynamic contact with irradiation
Ag/TiO_2_ [115]	20–40 nm spherical	*E. coli*, *P. aeruginosa*, *C. albicans*	agar well diffusion
TiO_2_ [38]	345 nm hollow spheres	*E. coli*, *E. coli MR 33.1*, *S. aureus MR 97-7 MR622-4*	dynamic contact
NiO [116]	18.6 nm spherical	*S. aureus*, *E. Coli*, *P. aeruginosa*	agar disk diffusion
NiO–ZnO/TiO_2_ nanotubes/Ti [4]	140–210 nm nanotubes	*E. coli*, *C. albicans*	dynamic contact with irradiation
NiO [117]	10–20 nm pleomorphic	*P. aeruginosa*, *E. coli MS-2 and MS-6*, *MR-10 and MR-31*	agar well diffusion
NiO [118]	22 nm grains	*A. niger*	agar well diffusion
NiO co-doped with B and N [119]	20.3 nm spherical	*E. faecalis*, *E. coli*	dynamic contact
CuO [120]	7–14 nm spherical	*E. coli*, *S. aureus*	agar disk diffusion
CuO and Cu_2_O [121]	sphere, rod and wire-shaped	*Klebsiella*, *S. aureus*	agar well diffusion
Ag/CuO [122]	38–57 nm	*E. coli*, *K. pneumonia*, *S. aureus*	agar disk diffusion
CuO [123]	10 nm rod and wire-shaped	*E. coli, S. flexneri, S. aureus*	agar well diffusion
ZnO–CuO [124]	500 nm rugged rod-like	*S. mutans*	dynamic contact

Copper nanoparticles are nanomaterials with good chemical stability, heat resistance, and excellent antimicrobial properties due to a large surface-area-to-volume ratio. Their excellent antibacterial, antifungal, antiviral, and anti-inflammatory properties prompted their application in many areas, such as food packaging and pharmaceutical industries [5,125–126]. Porta et al. (2019) obtained spherical Cu NPs through chemical reactions and these NPs have a strong bactericidal effect against Gram-negative and Gram-positive bacteria [108]. Composites containing Cu NPs and ZnO were developed by deposition of needle-like and spherical Cu NPs on a ZnO surface. These composites were exposed to visible light radiation in order to determine the minimum inhibitory concentration (MIC) and minimal bactericidal concentration (MBC) against *S. aureus* and *E. coli.* The composites exhibited antibacterial activities with low MIC and MBC values for *E. coli* (250 µg/mL and 750 µg/mL, respectively) and *S. aureus* (250 µg/mL and 500 µg/mL*,* respectively) [106]. Kolb et al. (2016) used the atmospheric pressure jet plasma method to deposit Cu NPs over acrylonitrile butadiene styrene substrates, generating potential antibacterial surfaces against *S. aureus* [104].

On the other hand, metal oxide nanoparticles are inorganic nanomaterials which have also presented relevant antimicrobial properties against several pathogenic microorganisms. Zinc oxide, titanium dioxide, copper oxide, and nickel oxide are the most typical metal-oxide NPs with potential antibacterial, antifungal and antiviral activities [127–128]. These oxides have been applied in the food packaging industry and also in the medical field, as shown in Table 3. ZnO is a semiconductor metal oxide with significant antimicrobial properties that can be further improved when applied as a nanomaterial. ZnO NPs have potential application in food preservation as well as important antibacterial properties against drug-resistant bacteria due to their size, shape and surface-capping agents [129–130]. Emamifar et al. (2010) developed orange juice packages based on low-density polyethylene (LDPE) nanocomposites with ZnO NPs. This packaging material presented a significant reduction in the microbial growth rate of *Lactobacillus plantarum* for up to 112 days of storage [112]. Star-like ZnO NPs were synthesized by the facile molten salt method and used to prepare synthetic nanocomposites with 2 or 4 wt % of ZnO NP load. Nanocomposites with 4 wt % of ZnO NPs exhibited the best antibacterial activity against *Bacillus subtilis* and *Enterobacter aerogenes* bacteria [109].

Titanium dioxide is also an inorganic material that is widely used in several products, including cosmetics and orthodontic composites, due to its excellent whitening, photocatalytic, and antimicrobial properties [131–132]. When the size of titanium dioxide is reduced to the nanoscale (TiO_2_ NPs), its photocatalytic property is greatly improved, generating more reactive oxygen species (ROS). ROS damages bacterial cells, DNA chains, and other cellular structures through oxidative stress. Therefore, the use of TiO_2_ NPs has been directed towards water disinfection, food packaging in addition to their known use as a UV filter to prevent skin cancer [114]. Lopez de Dicastillo et al. (2019) developed hollow TiO_2_ nanotubes and nanospheres with high antimicrobial activity through the combination of electrospinning and atomic layer deposition techniques [38–39]. The results indicated that TiO_2_ nanospheres exhibited the best antimicrobial activity against methicillin-resistant *Staphylococcus aureus 97-7* and *622-4* when the NPs were irradiated with UVA radiation for 60 minutes, which increased their antimicrobial properties in comparison to commercial TiO_2_ NPs [38]. Youssef et al. (2018) developed a biocomposite in the nanoscale with 2 wt % of TiO_2_ NPs which prevented mold growth on a cheese surface during an antimicrobial assay [3].

Nickel oxide nanoparticles have a multifunctional nature with interesting photocatalytic, electrochemical, and catalytic properties. Furthermore, NiO NPs exhibit anti-inflammatory properties, generating interest in the biomedical field to use these NPs as antibiotics or in cancer treatments [116,133]. NiO NPs synthesized from *Eucalyptus globulus* leaf extract showed excellent antibacterial activities against *E. coli*, *P. aeruginosa*, methicillin-sensitive and resistant *S. aureus* [117]. Suganya et al. (2018) developed a potent antifungal nanocomposite with NiO NPs against the *Aspergillus niger* strain. The authors attributed the excellent antifungal properties to the physical process used to internalize the powdered nanomaterial in the fungi cells and also to the chemical process that involved ROS generation [118].

Copper oxide is a metal oxide with the ability to target various bacterial structures and its antimicrobial activity can be further improved on the nanoscale. Due to their excellent properties, CuO NPs have attracted great interest from the healthcare, food packaging, medical, and environmental industries [120,134]. This metal oxide is capable of disrupting the normal function of the cell membrane, changing its permeability and the cellular respiration process [135]. Matsuda et al. (2019) have developed a fluoride-containing ZnO–CuO nanocomposite which inhibited the bacterial growth of *Streptococcus mutans,* showing a potential use in dental materials [124]. CuO nanorods (110 nm in length and 10 nm in diameter) were obtained by the precipitation process. The antimicrobial studies revealed good antimicrobial activity against *E. coli*, *S. flexneri*, and *S. aureus* cells [123].

#### Superparamagnetic iron-oxide nanoparticles

Superparamagnetic iron oxide nanoparticles are a special class of metal-oxide NPs with magnetic properties and excellent biocompatibility. Their shape, size and magnetic nature enables them to kill microorganisms through the application of an external magnetic field, resulting in an increase of the therapeutic antimicrobial properties, especially when compared to conventional antimicrobial compounds [136]. Ferromagnetic nanoparticles are probably the most known and studied SPIONs. Magnetite (Fe_3_O_4_) and maghemite (γ-Fe_2_O_3_) are two crystalline phases of iron oxide that present superparamagnetic properties at the nanoscale (<20 nm). This superparamagnetism is generated due to the reduced size of these nanoparticles which allow for a higher surface-to-volume ratio, increasing the surface of the atoms [19]. In addition, when a magnetic field is applied the magnetic moments of these ferromagnetic FeO NPs become aligned.

The surface of the SPIONs can be modified to specifically improve their functionality as antimicrobial compounds by increasing their interaction with the bacterial cells [137]. For example, chemical groups can be grafted onto and metals can be adhered to these NPs. Mahmoudi and Serpoooshan developed silver-ring-coated SPIONs through the coating of monodispersed SPIONs with carboxylated dextran via the ligand exchange method followed by conjugation with ethanediylbis(isonicotinate), which allowed for the chelation of the metal ions. These SPION silver core–shell NPs with clear ligand gaps and magnetic properties have the ability to absorb metallic NPs on their outer surface at a high packing density, which significantly enhances their properties [138].

Another strategy in which SPIONs are used to inhibit and/or reduce microbial incidence in biological and environmental applications is through the application of weak magnetic fields [139]. Park et al. demonstrated a 4 log inactivation of *Pseudomonas aeruginosa* through local heating created by using a 60 mg mL^−1^ SPION solution and applying an alternating current for 8 min.

#### Silica- and carbon-derived nanoparticles

Over the last years, several studies have revealed that silica nanoparticles are excellent antimicrobial metal-releasing systems due to their high chemical and thermal stability and good biocompatibility [20]. The Si NPs enhance the bactericidal effects of some compounds, mainly metallic systems, against a broad range of microorganisms due to their easy delivery [20,140]. In addition, their surfaces can be easily modified by relatively inexpensive precursors which can increase their efficiency. Bactericidal properties of nitric-oxide-releasing Si NPs against *P. aeruginosa*, *E. coli*, *S. aureus*, *S. epidermis*, and *Candida albicans* were studied and the results indicated an increased antimicrobial effectiveness due to a greater amount of nitric oxide released by the Si NPs [141]. In addition, the antimicrobial efficiency of silver and copper nanoparticles have also been improved through the development of Si–Ag and Si–Cu NPs. This achievement is specifically important when NPs present cytotoxic effects. Silver NPs were immobilized on hollow silica nanospheres or nanotubes which increased their antimicrobial activity at lower Ag NP concentrations. This is an effect of the morphology of the tubular hollow structures which present a better retention of Ag NPs [142]. Maniprasad and Santra (2012) developed novel core–shell silica structures containing highly dispersed Cu NPs. The bioavailability of these antimicrobial NPs had lower MIC values against *E. coli* and *B. subtilis* than copper hydroxide particles in suspension [143].

Silver carbon complexes with different formulations, including micelles and NPs, have also shown an antimicrobial effect since they inhibit the growth of some specific pathogenic bacteria, such as *P. aeruginosa* (–), *Burkholderia cepacia* (–), and *Klebsiella pneumoniae* (–), and antibiotic-resistant bacteria, such as *S. aureus* and *Acinetobacter baumannii* (–) [144].

### Antimicrobial action of metal-based nanoparticles

#### Methods for evaluating in vitro antimicrobial activity

**Figure 2:**
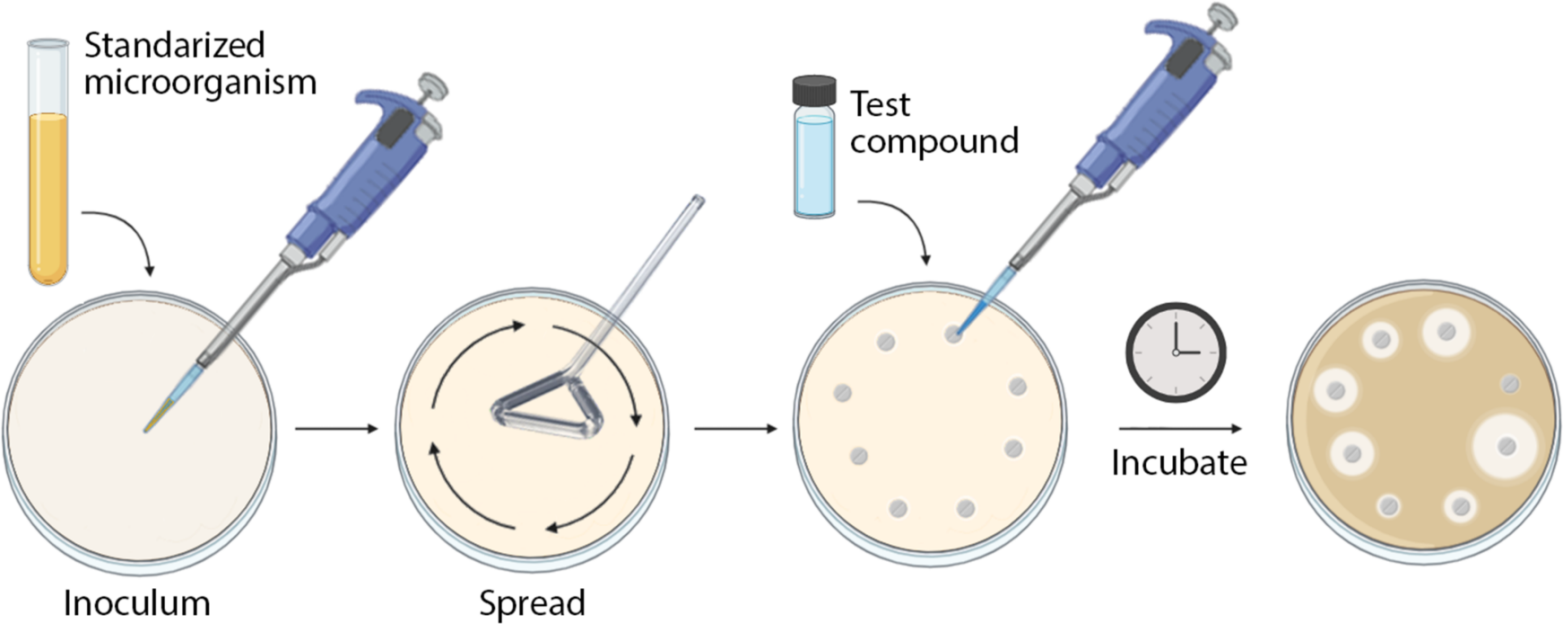
Scheme of the agar disk diffusion method. (Created with BioRender.com. Reproduction of this figure requires permission from BioRender.com).

The in vitro antimicrobial activity of metal-based NPs can be evaluated through several clinical microbiological methods, where the disk diffusion and the broth or agar dilution methods are the main techniques used. The agar disk diffusion method is routinely used to analyze the growth of common microorganisms, such as bacteria, fungi, and yeast, in a rapid manner. As Figure 2 shows, in this method, a standardized concentration of the microorganism is inoculated onto the Petri dish containing the growth culture medium, and filter paper disks with the antimicrobial agents are placed on the agar surface. The Petri dishes are incubated under appropriate growth conditions. The antimicrobial agent can be spread on the agar plate and it inhibits the microorganism growth by forming disks corresponding to inhibition zones [145].

**Figure 3:**
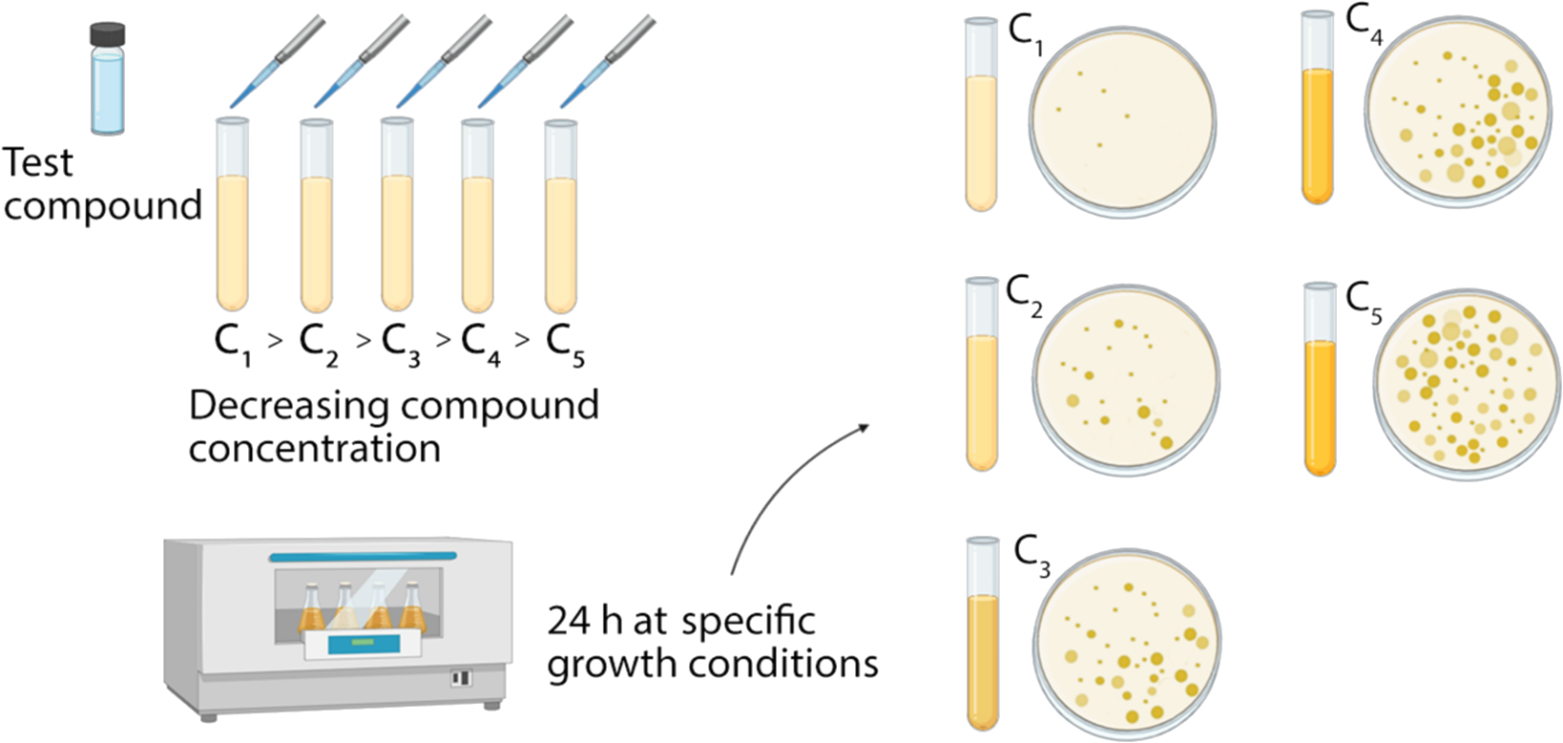
Scheme of the agar dilution method. (Created with BioRender.com. Reproduction of this figure requires permission from BioRender.com).

A standardized methodology in which the measurements of the inhibition zone diameter can be correlated with the minimum inhibitory concentrations of the antimicrobial agents needs to be used in order to obtain reliable results. Thus, specific culture media, various incubation conditions, and interpretive criteria for the inhibition zones are used. As a result, approximate MIC values can be obtained; however, this method cannot distinguish between bactericidal or bacteriostatic effects [146]. The agar diffusion method can also be modified depending on the research study. For example, in order to study the antibacterial activity of Ag NPs (synthesized by using the *Papaya carica* latex extract as the reducing agent) against different pathogenic bacteria, such as *Bacillus subtilis*, *Enterococcus faecalis, Escherichia coli*, *Vibrio cholerae*, *Klebsiella pneumonia*, and *Proteus mirabilis* the agar diffusion method was specifically modified. The modifications included substituting the filter paper disks for wells made in the Petri dish agar which were filled with different concentrations of Ag NPs. After the incubation period, the inhibition zones were measured. This alternative is generally called the agar well diffusion method. The MIC values of Ag NPs against each bacteria were reported and their highest antibacterial effect was against Gram-negative bacteria [10]. A second widely used method to measure antimicrobial activity is the agar (or broth) dilution method. This method consists of preparing a series of plates (or tubes) containing a standardized suspension of the microorganism to be tested into agar (or broth medium), containing various concentrations of the antimicrobial agents (Figure 3).

After incubation under the appropriate conditions, the MIC can be determined and the results can be analyzed using approved cutoff points. When using this technique, the experimental conditions must be carefully controlled in order to achieve reproducible results [147]. This technique is usually combined with the dynamic contact methodology (ASTM E2149-10 directive) in which different NP concentrations are put into contact for a given time period with a solution containing a known concentration of microorganisms. Therefore, after the NPs perform their antimicrobial activity in the liquid culture medium, it can be further inoculated onto the Petri dish with agar and incubated at the specific growth conditions, according to the target microorganisms [145,147–148].

In general, both mentioned methodologies are the most common techniques used. In case more information is needed regarding the inhibitory effect (bactericidal or bacteriostatic) or the cell damage caused by the NPs against the target microorganism, dead time tests and flow cytofluorometry methods can also be performed, among other tests [146].

**Figure 4:**
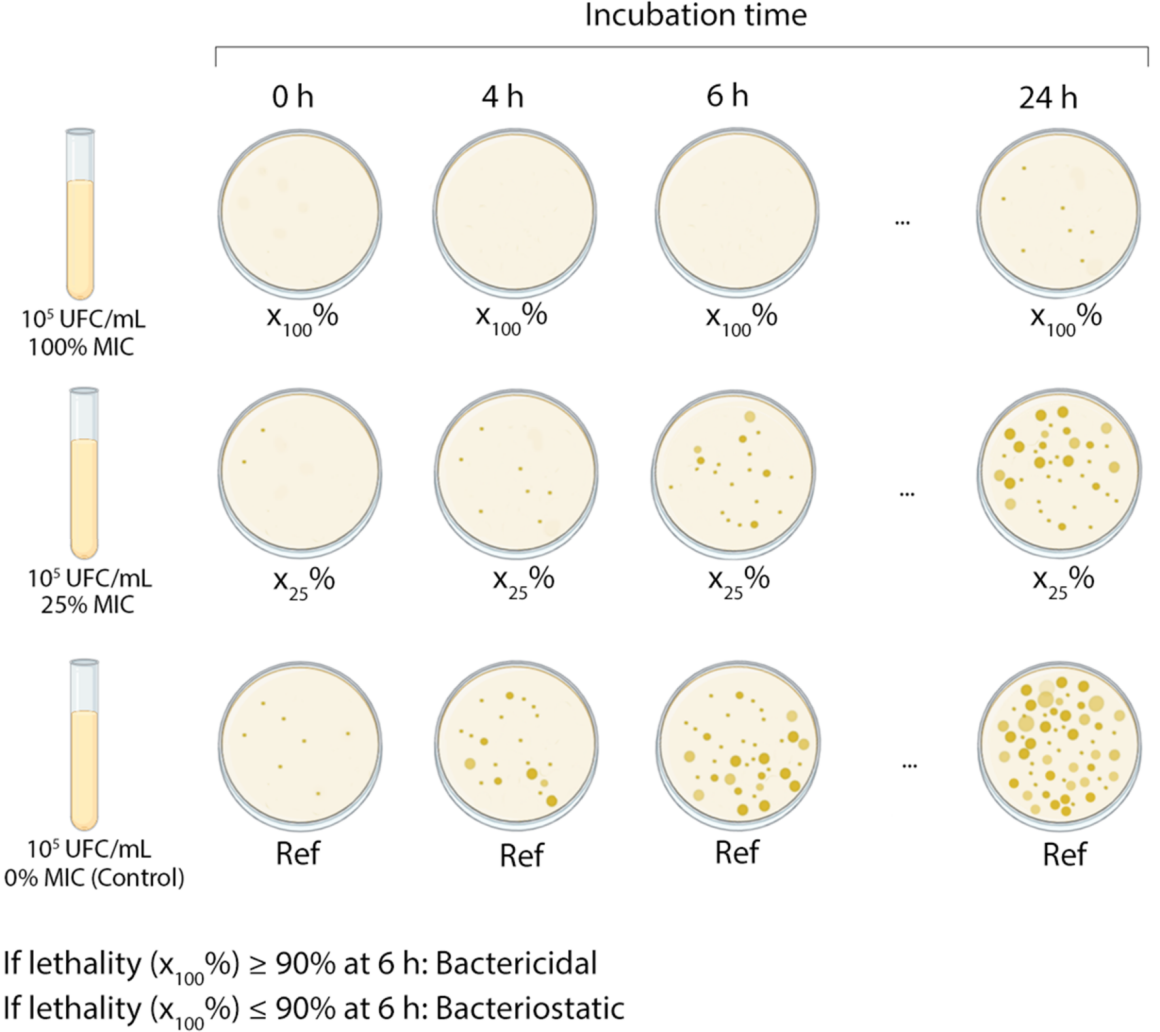
Scheme of dead time test. (Created with BioRender.com. Reproduction of this figure requires permission from BioRender.com).

The dead time test or time-kill test (Figure 4) can have different configurations depending on the test aim. In this method, the microorganisms are incubated in the presence of the antimicrobial compound at different incubation times (0, 2, 4, 8, 10, 12, and 24 h). The percentage of surviving bacteria, with respect to a control sample without the antimicrobial agent, is determined. A kill curve can be determined with the collected data in order to visualize the kinetics of the antimicrobial agent and to determine if it performs a bactericidal or bacteriostatic effect under certain established criteria (Figure 4). The variations in this method can determine the synergism or antagonism between two or more antimicrobial compounds, according to a ≥2 log difference in the antimicrobial activity between the compounds used, and the best constituent can be determined after 24 h of incubation [149].

The cytofluorometry method is an advanced and important technique that allows for the accurate identification of a cell population with fluorescence tagging by using a flow cytometer and a photodetector [150]. In comparison to other techniques, this method requires expensive equipment and, therefore, it is more commonly used in medical analysis and clinical medicine. With the techniques mentioned earlier, it is possible to determine significant differences between a wide variety of antimicrobial compounds against common food pathogens in a simpler way without major equipment requirements.

#### Mechanisms of antimicrobial action

The exact antibacterial mechanisms of NPs are being exhaustively investigated and some processes have been elucidated, including oxidative stress induction, metal ion release, and non-oxidative damage, which affect different structures from different microorganisms.

**Figure 5:**
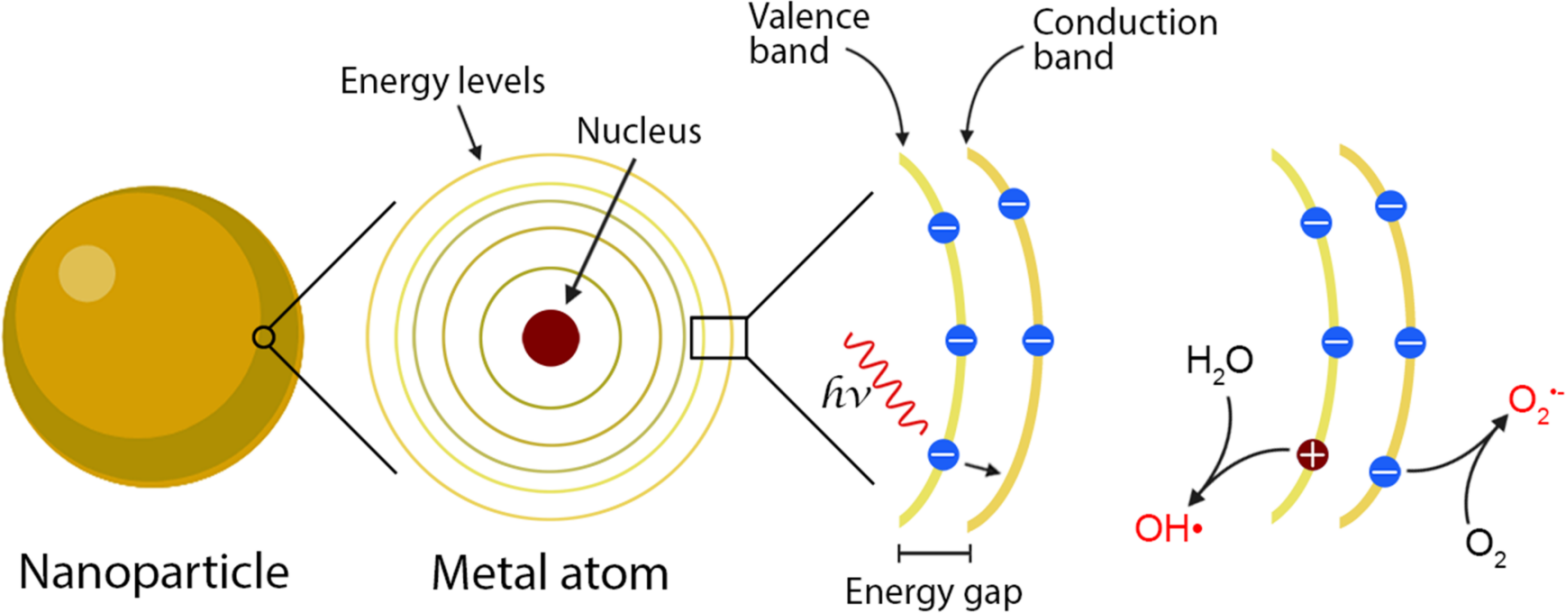
ROS production mechanism from the nanoparticles. (Created with BioRender.com. Reproduction of this figure requires permission from BioRender.com).

Reactive oxygen species are a group of molecules (or reactive intermediates) that even though they exist in nature for a short period of time (half-life varying between 10^−9^ and 10^−3^ s) they have a great oxidative potential that can eventually be toxic to microorganisms [151]. Superoxide radicals (O_2_^−^), hydroxyl radicals (•OH), hydrogen peroxide (H_2_O_2_), and singlet oxygen (^1^O_2_) are the most well-known ROS. The mechanism that better explains the synthesis of ROS from NPs is based on their photocatalytic activity (Figure 5). Metal compounds receive enough energy from light irradiation to excite and mobilize an electron from the valence band to the conduction band, leaving a highly reactive gap (H^+^). This zone becomes a ROS source as it interacts with H_2_O or OH^−^ that surrounds the nanoparticles [152].

In addition to molecules such as ascorbic acid, carotene, and tocopherol, microorganisms have an enzymatic antioxidant defense system, including catalase and superoxide dismutase (SOD), which controls the oxidative stress, reducing lipid peroxidation and the effects of ROS radicals, such as OH_2_^•−^ and OH^•^. At normal aerobic microorganism conditions, the production and clearance of ROS in cells are balanced by those enzymatic systems. Nevertheless, when these reactive species are in excess, a set of redox reactions can lead to cell death by the alteration of different essential structures (such as cell membrane, DNA, proteins, and electron transport chain) and metabolic routes which are responsible for maintaining the normal morphological and physiological cellular functions [153].

In addition to the oxidative stress, released metal ions from the metal oxide NPs can spread through the cell membrane into the cytoplasm and organelles. Metallic ions can interact with the functional groups of proteins and nucleic acids, such as thiol (–SH), amino (–NH), and carboxyl (–COOH) groups, and therefore, might affect the enzymatic activities and several protein structures. Although the metal ions released are not the main source of damage caused by NPs, it is important to mention that some authors have identified them as good carriers of other antimicrobial molecules, improving their transport to the target [154], which offers protection against resistance by the target bacteria, and facilitates the permeation through the cell membrane. Metal ions can also allow for the combination of multiple antimicrobial agents in the same NP in order to improve their effects and overcome resistance mechanisms, such as the efflux pump systems.

The absence of lipid peroxidation biomarkers and a small amount of metal ions detected by energy-dispersive X-ray spectroscopy in bacteria in the presence of MeO NPs have confirmed that oxidative damage and metal ion release are not exclusive antimicrobial mechanisms [155]. Critical cellular processes related to the proteins, including amino acid, carbohydrate, and nucleotide metabolisms, are significantly reduced, leading to cell death.

The combination of oxidative stress, metal ion release, and non-oxidative damage affects cell structures upon NP exposure in several ways. In the following sections, these cell damage cases will be briefly explained.

#### Cell wall damage

The bacterial cell wall provides rigidity, shape, and protection to the cell against osmotic rupture and mechanical damage. It is the first barrier against any harmful particles from the environment, such as oxidative molecules. Every type of microorganism has a different cell wall composition: i) fungi and yeast are mainly composed of chitin and polysaccharides; ii) Gram-positive bacteria contain many layers of peptidoglycan and teichoic acid (20–50 nm); and iii) Gram-negative bacteria present a few layers of peptidoglycan surrounded by a second lipid membrane containing lipopolysaccharides and lipoproteins [156–157]. Therefore, the cell wall damage caused by NPs can occur through different processes.

Many studies have shown that NPs present better activity against Gram-positive bacteria in comparison to Gram-negative bacteria. The presence of negative charges, given by the lipopolysaccharides in the outer membrane in Gram-negative bacteria, slightly attract NPs [158]. In addition, the double membrane acts as a selective physical barrier against hydrophobic compounds, such as detergents and antibiotics. On the other hand, Gram-positive bacteria have a higher permeability, even with a thick layer of peptidoglycan, since the single membrane is not enough to avoid the entrance of foreign molecules. Besides, the cell wall has a higher negative charge than Gram-negative bacteria [159], given by the characteristics of peptidoglycan and teichoic acid structures which strongly attract NPs, resulting in cell membrane damage and cell death [160]. Silver, gold, zinc oxide, and titanium dioxide NPs can be attracted to the cell wall by electrostatic attraction [161], van der Waals forces [162], and hydrophobic interactions [163], inducing changes in the shape, function and permeability of the cells.

#### Proteins and DNA

**Figure 6:**
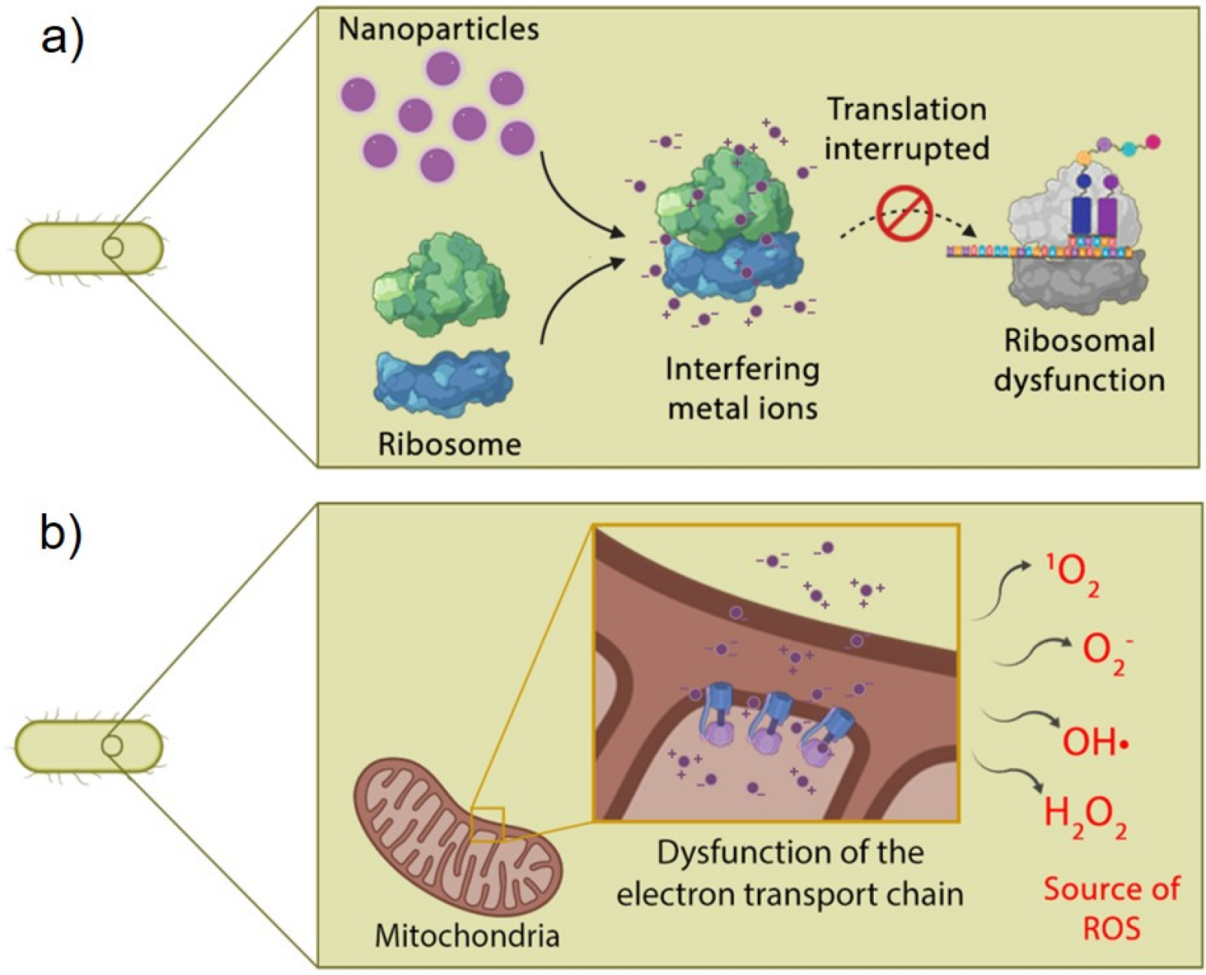
a) Ribosomal dysfunction caused by metal-ion-containing nanoparticles. b) Dysfunction of the electron transport chain caused by metal ions of the nanoparticles. (Created with BioRender.com. Reproduction of this figure requires permission from BioRender.com).

Proteins play a fundamental role in microorganism-catalyzing metabolic reactions and are a fundamental part of cellular structures. Proteomic analysis has revealed deregulation in proteins involved in nitrogen metabolism, electron transfer, and substance transport in the presence of CuO NPs [164]. Silver ions released from Ag NPs can affect the expression of the ribosomal subunit that interacts with sulfur- and phosphorus-containing groups of proteins, even in the cell wall and plasma membrane bacteria [165–166]. Cui et al. (2012) showed that Au NPs prevented the combination of a ribosomal subunit with tRNA and collapsed the membrane potential (Figure 6a), inhibiting the ATPase activity. This, in turn, reduced the ATP levels and stimulated the generation of ROS, simultaneously affecting other structures (Figure 6b) [167].

**Figure 7:**
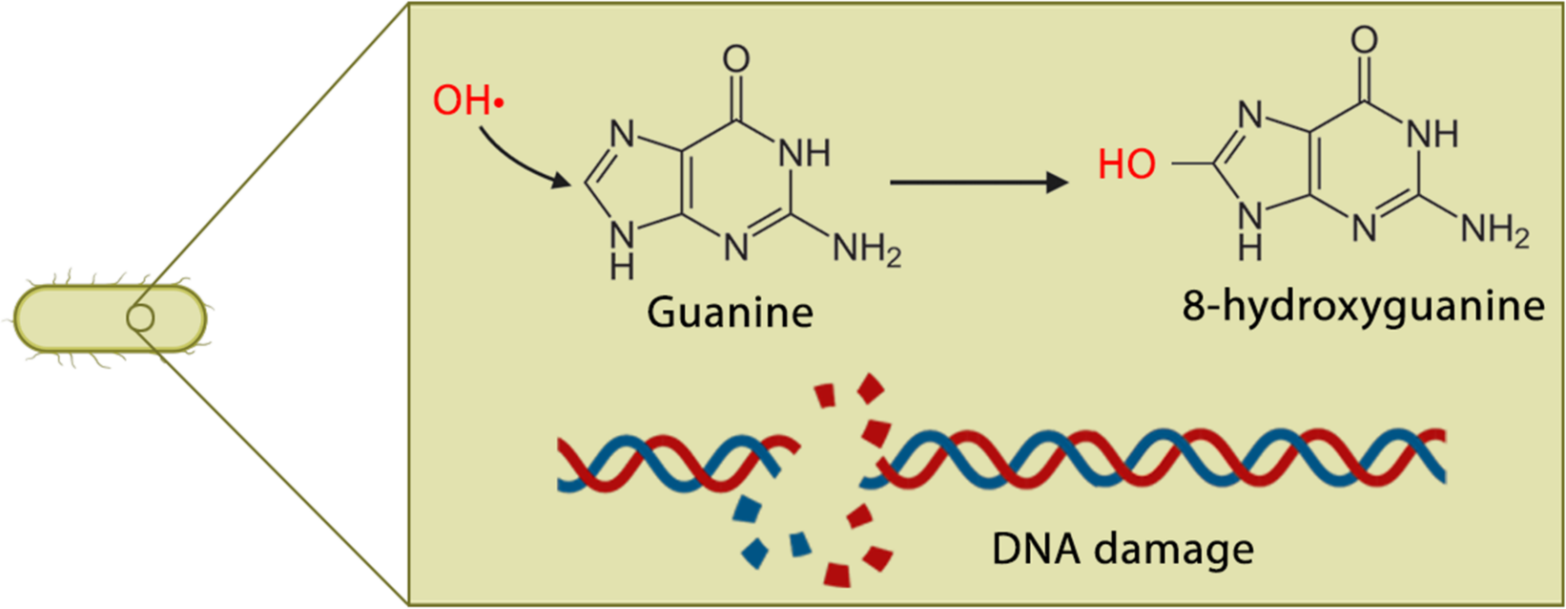
DNA damage caused by ROS. (Created with BioRender.com. Reproduction of this figure requires permission from BioRender.com).

Genomic analysis has shown that TiO_2_ NPs can affect regulatory microorganism metabolic replication, transcription, and cell division since ROS can generate DNA mutations. These modifications may target the sugar-phosphate or the nucleobases and cause saccharide fragmentation and strand break [160]. This cleavage induced by the nanoparticles was studied in the pBR322 plasmid in the presence of Ag NPs via electrophoresis [168]. The results showed that guanine is the most affected nucleobase due to its low redox potential, and its oxidation produces a wide variety of modifications that ultimately affect DNA function (Figure 7) [169]. NPs can not only affect bacteria but also other complex multicellular organisms through the induced genetic damage [170].

#### Changes in expression of metabolic genes

Every enzymatic detoxification system (e.g., SOD, glutathione and catalase) is regulated by a signal which senses the ROS level and changes the expression level of certain set of genes in a way to protect against and minimize the oxidative damage. In addition, microorganisms can modify metabolic routes and redirect resources to repair and reinforce damaged structures, such as the cell membrane or the DNA itself, through the overexpression of genes related to those functions. In *Escherichia coli* and *Pseudomonas putida*, genes related to the general stress response were upregulated. Genes protecting against hydrogen peroxide oxidative damage, catalase/hydroperoxidase, superoxide radicals degradation genes, superoxide dismutase, and superoxide removal transcriptional activator, were upregulated in a range varying from 3.2-fold to 9.2-fold after a 2 h incubation period with Ag NPs [171].

## Conclusion

The research related to the development of novel antimicrobial nanoparticles is significantly relevant nowadays. In this review, the main issues regarding antimicrobial nanoparticles, including their synthesis techniques, types, characterization of their properties, and their antimicrobial mechanisms, are discussed.

Different methods used for obtaining nanoparticles based on the traditional physical and chemical procedures have been compared, as well as the most innovative technologies based on the so-called green synthesis method, which is attracting much attention lately due to its reduced environmental impact. Most nanoparticles are based on metal and metal-oxide compounds, and the strategies used to control their delivery and to increase their antimicrobial activity have been related to the use of silica nanoparticles in their manufacturing process. Although the oxidative stress is the main mechanism by which the nanoparticles can eliminate microorganisms, other processes may be intimately related to the NP antimicrobial activity.

The information presented in this work encourages the search for new nanomaterials with controlled morphology and dimensions. In addition, synthesis processes that allow nanomaterials to be processed in a control manner and result in ecologically friendly materials were highlighted. In addition, this review highlights the need for further investigation into the possible mechanisms of these nanoparticles and the development of new substances with high antimicrobial activity.

## Future Perspectives

The generation of reactive oxygen species is the main mechanism by which nanoparticles can trigger antimicrobial activity, the degree of which can vary depending on their material, morphology, and size. This antimicrobial activity can be used in numerous sectors, such as textile, animal, or antimicrobial packaging industries. In the latter, NPs are used to inhibit and control microbial growth, resist against the penetration of liquids or gases, retain moisture, and maintain packaged food shelf life. The global market for antimicrobial materials and packaging has demonstrated significant growth, which leads us to think that there will be a strong increase in the demand for nanoparticles with antimicrobial properties in which synthesis processes can be industrially scaled. In order to combat nosocomial infections (which are a current and urgent worldwide problem), another potential application of metal-based nanoparticles with antimicrobial activity is the coating of the surfaces of noncritical equipment in medical care facilities in order to create antimicrobial surfaces.

At this point, it must be emphasized that microorganisms are becoming increasingly resistant to disinfectants as well as to traditional antibiotics, and nanoparticles with antimicrobial properties can be an effective complement in the fight against these pathogenic microorganisms. However, it is essential to keep in mind that it is not only important to develop potential applications for antimicrobial NPs, but also to follow safety regulations that allow for the control of inhalation, migration, skin penetration, and ingestion of nanoparticles, which could potentially induce human health issues. It should also be kept in mind that metal-based nanoparticles are toxic to many organisms at high concentrations and discarding these NPs in the environment can introduce serious environmental problems.
